# Fabrication of Silicon Carbide Fiber-Reinforced Silicon Carbide Matrix Composites Using Binder Jetting Additive Manufacturing from Irregularly-Shaped and Spherical Powders

**DOI:** 10.3390/ma13071766

**Published:** 2020-04-09

**Authors:** Igor Polozov, Nikolay Razumov, Dmitriy Masaylo, Alexey Silin, Yuliya Lebedeva, Anatoly Popovich

**Affiliations:** 1 Peter the Great St. Petersburg Polytechnic University, Polytechnicheskaya, 29, 195251 St. Petersburg, Russia; n.razumov@onti.spbstu.ru (N.R.); dmasaylo@gmail.com (D.M.); silin8888@mail.ru (A.S.); director@immet.spbstu.ru (A.P.); 2 Federal State Unitary Enterprise “All-Russian Scientific Research Institute of Aviation Materials” State Research Center of the Russian Federation, 17 Radio str., 105005 Moscow, Russia; yulia.ananieva@gmail.com

**Keywords:** additive manufacturing, binder jetting, silicon carbide, spray drying, pyrolysis

## Abstract

In this paper, silicon carbide fiber-reinforced silicon carbide (SiC_f_/SiC) composites were fabricated using binder jetting additive manufacturing followed by polymer infiltration and pyrolysis. Spherical SiC powders were produced using milling, spray drying, and thermal plasma treatment, and were characterized using SEM and XRD methods. Irregularly shaped and spherical SiC powders were used to obtain SiC_f_/SiC blends for the application in binder jetting. The effect of SiC powder shape on densification behavior, microstructure, and mechanical properties of binder jetted SiC_f_/SiC composites was evaluated. The highest density of 2.52 g/cm^3^ was obtained after six polymer infiltration and pyrolysis cycles. The microstructure and mechanical properties of the fabricated SiC_f_/SiC composites were characterized. Using the spherical SiC powder resulted in higher fracture toughness and hardness, but lower flexural strength compared to the irregularly shaped powder. It was shown that it is feasible to fabricate dense SiC_f_/SiC composites using binder jetting followed by polymer infiltration and pyrolysis.

## 1. Introduction

Silicon carbide fiber-reinforced silicon carbide (SiC_f_/SiC) ceramic matrix composites (CMC) are considered to be promising materials for advanced applications in aerospace engines, gas turbines, and nuclear reactors [1]. Several methods have been used to make SiC_f_/SiC particulate-based composites with pressure-assisted methods such as hot pressing, as well as pressed preforms that can be post-processed with sintering, liquid silicon infiltration (LSI), chemical vapor infiltration (CVI), and precursor impregnation and pyrolysis (PIP) [2,3,4,5,6]. The main limitation of these methods is the inability to produce parts with complex geometries. Machining of ceramics is an expensive and labor-consuming process; thus, achieving net shaped parts is important for saving costs. Often, up to 80% of the cost of manufacturing of ceramic parts may reflect the mechanical tooling cost [7].

Additive manufacturing (AM) offers a possibility to produce complex structures from various materials [8,9]. At the moment, the majority of AM processes, excluding those used for manufacturing parts of polymers, use a laser or an electron beam as the energy source, and metal powder or wire as the feedstock material. Such an approach limits the number of materials used in AM, in particular, for intermetallic and ceramic materials. At the same time, demand for ceramic parts for the aerospace and energy industries has been constantly increasing [10].

Binder jetting technology, which utilizes powders as feedstock material, is one of the methods for AM of ceramic components, as Gonzalez et al. [11] and Du et al. [12] showed using alumina powders. Binder jetting involves depositing a binder on a powder layer and curing the binder to obtain a green part [13]. One of the major drawbacks of this technology is the high porosity and poor mechanical properties of the produced green parts [14,15]. This results from low powder packing density in an applied powder layer as well as the use of a binder to build a part, which leads to a weak connection between powder particles as opposed to other AM process.

Stereolithography and robocasting are other AM methods that can be used to fabricate ceramic parts [16,17]. These methods require the preparation of colloidal gels and photocuring slurries, which might be time-consuming. Moreover, SiC exhibits a high refractive index and light absorption in the ultraviolet wavelength, which makes it challenging for the stereolithography [18].

Even though the parts obtained by binder jetting are characterized by a high level of porosity, it is still possible to achieve a high density of ceramic parts after subsequent sintering or infiltration. For example, Gonzalez et al. [11] achieved 96% of the theoretical density for aluminum oxide parts after sintering at 1600 °C for 16 h. Fielding et al. [19] achieved 95% density after sintering by adding ZnO and SiO_2_ to (Ca_3_(PO_4_)_2_) powder. Melcher et al. [20] infiltrated alumina green samples having 36% density using copper to fabricate dense parts. A similar process was used to infiltrate alumina samples with glass by Zhang et al. [21]. Another example of such an approach is the production of reactive-bonded ceramic based on SiC; Fu et al. [22] used a mixture of silicon, silicon carbide, and dextrin to obtain samples by binder jetting followed by pyrolysis and liquid silicon infiltration, which resulted in reactive-bonded silicon carbide parts. However, such an approach limits the maximum working temperature, since silicon has a melting point of 1414 °C. Moon et al. [23] fabricated a carbon green-part by binder jetting and then infiltrated it with silicon, which resulted in the formation of a reactive-bonded SiC-based composite material. Monolithic SiC samples with >90% of theoretical density have been fabricated by Terrani et al., by combining binder jetting and chemical vapor infiltration processes as shown in [24]. Fleisher et. al. [25] used binder jetting followed by phenolic resin binder impregnation and capillary liquid silicon infiltration to successfully fabricate complex-shaped SiC parts; however, residual silicon would limit the maximum operating temperature of such parts.

Even though the binder jetting process has been applied to fabricate parts from various ceramic materials, the studies devoted to SiC_f_/SiC fabrication are limited. In a previous paper [26], SiC_f_/SiC composites were fabricated using the binder jetting process followed by PIP. However, only irregular-shaped SiC powder particles were used. Spherical powders are considered to be preferable in AM processes, including binder jetting technology, since they provide better flowability, packing density, and sinterability compared to irregularly shaped particles [27,28]. Thus, it is important to investigate the effect of powder shape and morphology on the properties of binder jetted SiC_f_/SiC CMCs.

The main objective of this paper was to investigate the fabrication process of SiC_f_/SiC CMCs by binder jetting AM technology using powder compositions based on both irregular and spherical SiC particles with SiC fibers. The feasibility of spray drying and plasma treatment processes to fabricate spherical silicon carbide powder for AM applications was investigated. The effect of SiC powder shape on densification behavior, microstructure, and mechanical properties of binder jetted SiC_f_/SiC composites was evaluated.

## 2. Materials and Methods

Silicon carbide F320 grit powders (d_50_ = 38.3 µm) with irregular (non-spherical) shape and Si-TUFF^TM^ silicon carbide fibers with 100–300 µm of length and 7 µm of average diameter were used in the binder jetting process. The SiC fibers and SiC powders were blended in a tumbler mixture for 12 h to obtain a composite mixture.

Prior to spray drying, SiC F320 grit powders were milled in a jet-type mill Netzsch CGS10 using the following process parameters: gas pressure = 7 atm., classifier’s rotation speed = 17,000 rpm, powder feeding rate = 50 g/min. The selection of the milling parameters was based on preliminary experiments. The milled powder had the following particle size distribution: d_10_ = 0.7 µm, d_50_ = 1.4 µm, d_90_ = 2.5 µm.

Silicon metal powder (99.9%) up to 5 µm in size was mixed with the milled SiC powder prior to the spray drying to act as a binder during the subsequent plasma spheroidization.

The spray drying process of the 95 SiC-5 Si (wt.%) powder blend was carried out using a LPG-5 spray dryer (Changzhou Yibu Drying Equipment Co. Ltd, Jiangsu, China). The spray dryer operating principle is the following. Filtered heated air is uniformly fed as a spiral downflow to a drying chamber through an air dispenser. The liquid material is sprayed inside the drying chamber through a centrifugal disk pulverizer. Upon contact with the hot air, the fine droplets of the pulverized liquid are dried and form separate fine particles falling on the chamber floor. A 10% water solution of polyvinyl alcohol (PVA) was used as a binder with a 1:1 weight ratio of the powder to the binder. The air temperature during the spray drying was 170–180 °C. The pulverizing disk rotating frequency was varied between 15–50 Hz.

Plasma jet treatment experiments were carried out using TekSphero 15 plasma spheroidization equipment manufactured by Tekna Plasma Systems Inc. (Sherbrooke, Québec, Canada). The preliminary experiments were carried out to choose the following plasma treatment process parameters to obtain SiC spherical particles: plasma torch power = 15 kW, plasma gas (argon hydrogen mix) flow rate = 2.4 m^3^/h, powder feeding rate = 10–70 g/min.

The ExOne Innovent binder jetting printer and ExOne solvent binder were used to build samples of 10 × 7 × 70 mm^3^ in size. The following printing parameters were used: 4–7 mm/s recoat speed, 20 mm/s roller speed, 120 rpm roller rotation speed, 30 s dry time at 60 °C drying temperature, 100 µm layer thickness, and 60% binder saturation. The green parts were cured at 190 °C for 3 h. The binder jetting process parameters were preliminary optimized to achieve a steady fabrication process of the samples with a desired geometry.

Polycarbosilane with StarPCS^TM^ SMP-10 trade name manufactured by Starfire Systems (Schenectady, NY, USA) was used for infiltration of the green parts. The infiltration process was carried out in a vacuum chamber for 1 h by vacuuming the chamber with the samples and then introducing SMP-10 into the chamber so that it fully covered the samples.

After the SMP-10 infiltration, the samples were subjected to pyrolysis in a heating furnace at 1000 °C for 1 h with an argon flow. The heating rate was 10 °C/min. An intermediate dwelling of the samples at 500 °C for 30 min was done to remove the gases forming during the heating of SMP-10. These parameters were chosen based on the data showed by [26] for SMP-10 pyrolysis used for binder jetted SiC samples, as well as for SMP-10 pyrolysis of electrophoretic deposited SiC-samples [29].

A summary of the types of SiC powders and treatments used in the current study is presented in Table 1.

Three-point flexural tests were carried out according to ISO 17138:2014 “Fine ceramics (advanced ceramics, advanced technical ceramics)—Mechanical properties of ceramic composites at room temperature—Determination of flexural strength” with the samples of 7 × 10 × 70 mm^3^ size.

The hardness H_v_ and fracture toughness K_1C_ of the fabricated ceramic samples were measured using a Buehler VH1150 testing machine (Buehler, Lake Bluff, IL, USA) with a 1000-g load and 10 s of dwell time. The hardness values were calculated according to Formula (1):

(1) $$H_{v}=1.854\cdot \frac{g\cdot F}{{\left(2a\right)}^{2}}\cdot 10^{-3},$$

where *F* is the load, H; *a* is the length of a semi-diagonal of the indentation, µm; *l* is the crack length, µm; *g* is the acceleration of gravity (9.8 m/s^2^). 

Fracture toughness was calculated according to the formula for the Palmqvist crack model (2):

(2) $$K_{\mathrm{Ic}}=0.048{\left(\frac{l}{a}\right)}^{-1/2}{\left(\frac{H_{v}}{E\Phi }\right)}^{-2/5}\left(\frac{H_{v}a^{1/2}}{\Phi }\right),$$

where *E* is Young’s module (taken as 360 GPa in this study), Φ is 3; *H*_v_ is the hardness; *a* is the length of a semi-diagonal of the indentation, µm; *l* is the crack length, µm.

The fracture toughness measurement method with a Vickers indenter was suggested by Evans and Wilshaw [30] and then further extended by Niihara [31]. The equations for calculating the fracture toughness using the Vickers indenter method are based on a semi-empirical calculation between the indentation load and the length of the cracks coming from the corners of the indent. 

The phase composition was analyzed with a Bruker D8 Advance X-ray diffraction (XRD) (Bruker, Billerica, MA, USA) meter using Cu-Kα (λ = 1.5418 Å) irradiation. The microstructure investigation and powder morphology studies were carried out using TESCAN Mira 3 LMU scanning electron microscope (SEM) (Tescan, Brno, Czechia).

The fabricated samples were measured using a digital caliper with a resolution of 0.01 mm and the results were compared to the CAD-data.

## 3. Results

### 3.1. Powder Characterization

**Figure 1:**
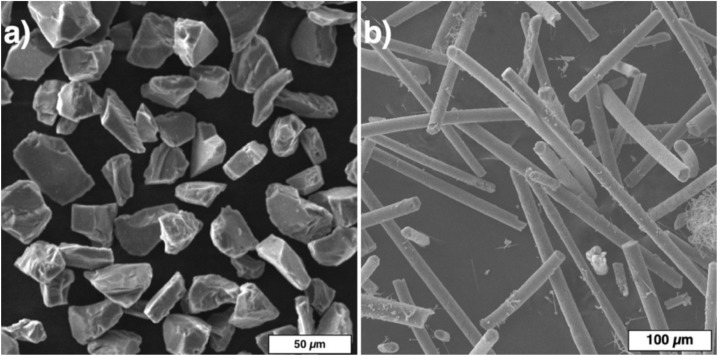
Initial silicon carbide (SiC) powder particles (**a**) and SiC fibers (**b**).

Figure 1 shows the initial SiC powder particles and the SiC fibers. The SiC powder features an irregular particle shape with smooth faces and has the following particle size distribution: d_10_ = 21.8 µm, d_50_ = 38.3 µm, d_90_ = 63.2 µm. The apparent density of the SiC powder is 1.37 g/cm^3^. The SiC fibers (Figure 1b) have a length of about 100–300 µm with a diameter of 7–10 µm.

**Figure 2:**
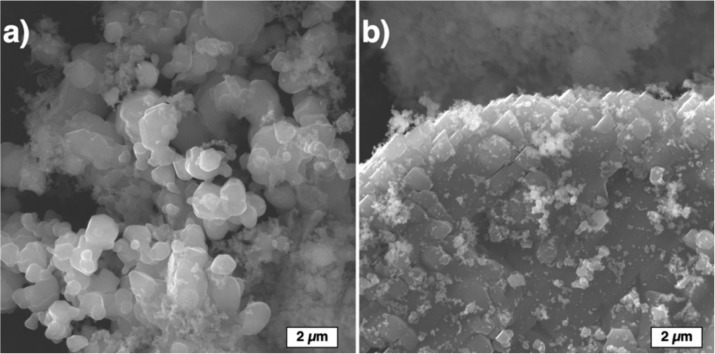
SiC F320 particles after treatment in a thermal plasma jet showing (**a**) condensated particles and (**b**) a particle’s surface morphology.

According to the Si-C phase diagram [32], SiC melts incongruently at 2830 °C and starts to decompose at about 1800 °C [33]. During its treatment in a thermal plasma jet, the initial powder partially sublimates followed by condensation of fine round particles with a size below 1 µm (Figure 2a). Epitaxial growth of columnar crystals also takes place at the surface of coarse particles (Figure 2b). Thus, a three-step approach has been suggested to obtain spherical SiC powder particles, which involves obtaining particles consisting of SiC, Si particles, and a binder using the spray drying process and then subjecting these particles to the plasma treatment.

**Figure 3:**
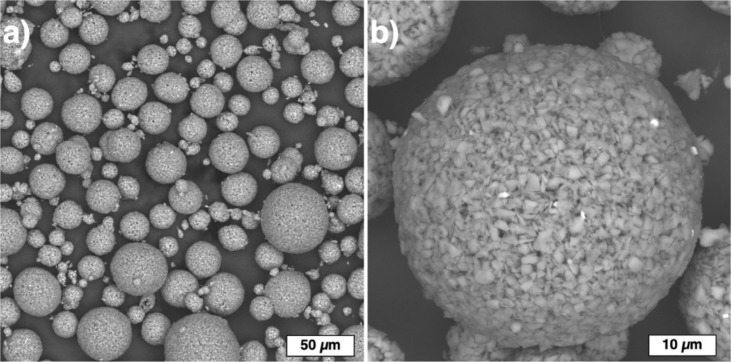
Powder particles obtained by spray drying of 95% SiC-5% Si slurry with polyvinyl alcohol (PVA) as the binder: (**a**) general view and (**b**) a particle’s surface morphology.

During the spray drying process, agglomerated spherical particles with the size 10–80 µm (Figure 3) were fabricated. The agglomerated particle surface consists of evenly distributed fine particles (Figure 3b). Two types of particles can be distinguished by their size: coarse particles (1–4 µm) and fine particles of submicron size. The fine particles fill the voids between the coarse particles.

**Figure 4:**
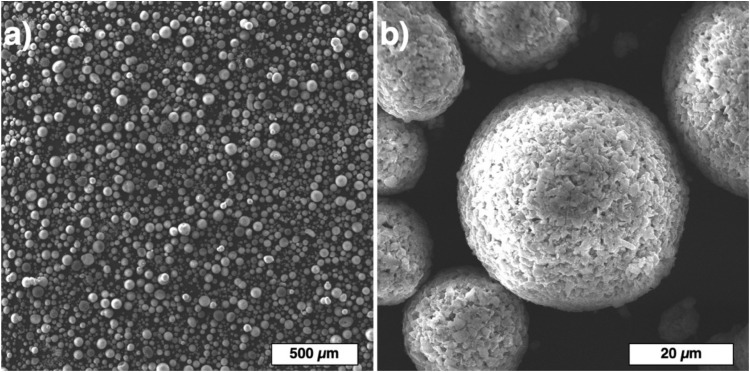
SiC powder particles after spray drying and plasma treatment: (**a**) general view and (**b**) a particle’s surface morphology.

Figure 4 shows the spherical powder particles obtained by the treatment of spray dried powders in a thermal argon hydrogen plasma jet. The obtained powder particles do not have internal voids and feature a homogeneous chemical distribution as confirmed by SEM investigations with back-scattered electrons. Local sintering of SiC particles occurs during the plasma treatment, which results in necking between the adjacent particles. During the plasma treatment, silicon melted and binded the SiC particles, while the binder dissociated with the formation of carbon. Carbon reacted with silicon and formed a reactive-bonded SiC. Similarly, the formation of β-SiC was reported in [34] when SiO and C powders were treated in a radiofrequency thermal plasma. The particle size distribution of the obtained powder after the plasma treatment was the following: d_10_ = 24.4 µm, d_50_ = 49.5 µm, d_90_ = 89.9 µm. The amount of powder particles below 10 µm was about 1.8% vol. The apparent density of the powder was 0.69 g/cm^3^, which is lower compared to the initial F320 powder due to finer SiC particles forming the spherical particles.

**Figure 5:**
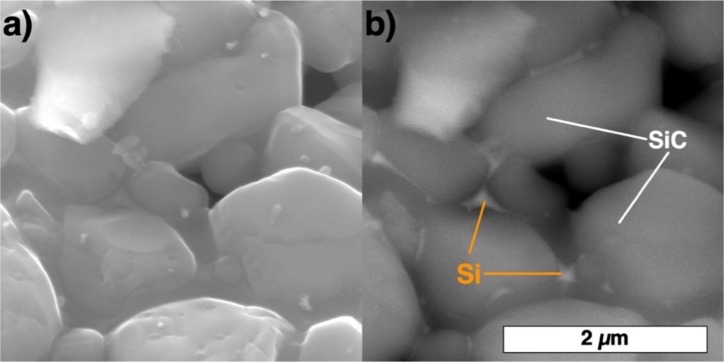
SiC powder particles after spray drying and plasma treatment in secondary electrons (**a**) and backscattered electrons (**b**).

As expected, during the plasma treatment of the spray dried powder, pure silicon was melted and bonded the SiC particles (Figure 5). The presence of the silicon was confirmed by the X-ray analysis illustrated below. At the same time, silicon partially reacted with the carbon formed from PVA dissociation, which resulted in the formation of the secondary SiC.

**Figure 6:**
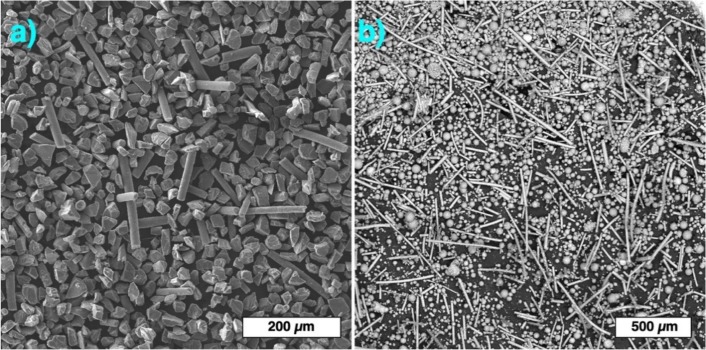
Composite blends prepared from SiC powders with irregular (**a**) and spherical (**b**) particles and 30% vol. of SiC fibers.

SiC powders mixed with 30% vol. of SiC fibers were used for application in the binder jetting process. Both irregular and spherical SiC powders were used to prepare the separate composite blends as shown in Figure 6.

### 3.2. Densification of the SiCf/SiC Samples During Infiltration and Pyrolysis

The binder jetting process was used to prepare the samples from the composite blends. The samples fabricated using irregular SiC particles had a bulk density of 1.34–1.39 g/cm^3^, while the samples obtained using spherical SiC particles had a bulk density of 0.72 g/cm^3^, as measured using the Archimedes method. The obtained density values correspond to 56–58% of porosity for the samples with irregular-shaped particles and 76% for the samples with spherical particles. High porosity values for the samples with spherical particles are due to the presence of submicron pores in the initial spherical SiC particles after the plasma treatment.

**Figure 7:**
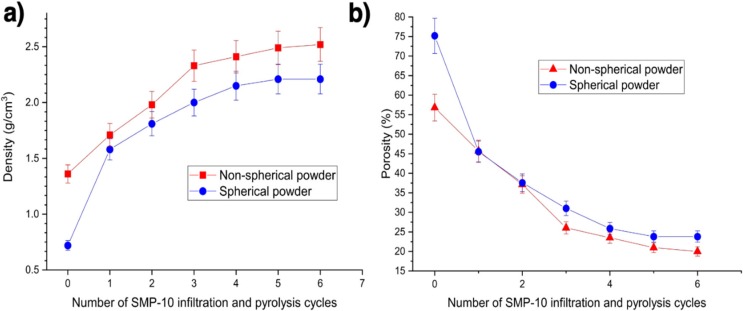
Change in density (**a**) and porosity (**b**) of the SiCf/SiC samples with the number of infiltration and pyrolysis cycles.

The fabricated green samples were subjected to several cycles of SMP-10 infiltration and pyrolysis. SMP-10 is known to have a good wetting ability that allows infiltrating very small pores [29]. During the pyrolysis process, SMP-10 polycarbosilane in the sample’s pores forms into secondary silicon carbide. Hence, the porosity of the samples decreases. Figure 7 shows the changes in density and porosity of the samples after various numbers of infiltration and pyrolysis cycles. After the first cycle, the density of SiC_f_/SiC samples, fabricated using irregular particles, increases from 1.36 g/cm^3^ to 1.71 g/cm^3^. The porosity decreases from 56% to 46%. For the samples fabricated using spherical particles, the density increases from 0.72 g/cm^3^ to 1.81 g/cm^3^, decreasing the porosity from 76% to 46%. The ceramic yield of the liquid polycarbosilane is approximately 60–70% [35,36]. Hence, a large volume fraction of pores still remains after the pyrolysis. While increasing the number of infiltration and pyrolysis cycles leads to higher density, the degree of impact decreases due to filling up the pores close to the surface of the samples with the secondary SiC and preventing the SMP-10 to further infiltrate the samples. Similarly, Halbig et al. [26] showed that the highest increase in density for binder jetted SiC samples occurred in the first two infiltration steps. After the sixth cycle, the density and porosity values for the sample fabricated from irregular particles reached 2.52 g/cm^3^ and 20%, respectively, while for the sample fabricated from spherical particles the density and porosity were 2.21 g/cm^3^ and 24%, respectively. A similar trend was reported in [36], when SiC_f_/SiC composites were fabricated by PIP of continuous SiC fiber preforms, however lower densities were achieved after the same number of PIP cycles.

### 3.3. Microstructure Characterization

**Figure 8:**
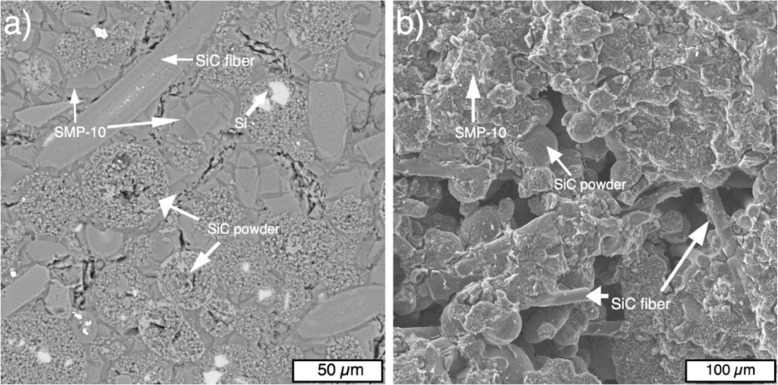
SEM images of the microstructure (**a**) and fracture surface (**b**) of the SiCf/SiC sample fabricated from spherical particles after six infiltration and pyrolysis cycles.

Figure 8 shows SEM images of the microstructure of the SiC_f_/SiC samples fabricated from spherical particles after six cycles of infiltration and pyrolysis (Figure 8a), and the fracture surface of the sample (Figure 8b). The microstructure features spherical SiC particles and separate SiC fibers with a small amount of Si particles. After several infiltrations and pyrolysis cycles, SMP-10 polycarbosilane formed a SiC phase, which resulted in partially closing the internal pores. The majority of the residual pores is located in coarse spherical SiC particles remaining from the initial particles. These pores are filled up during the first SMP-10 infiltration, which prevents further infiltration of these pores. The fracture surface of the samples features the initial separate powder particles of SiC and SiC fibers that are bonded by secondary SiC formed from SMP-10. There are also interconnected pores that might act as stress concentrators during the load and initiate crack formation.

**Figure 9:**
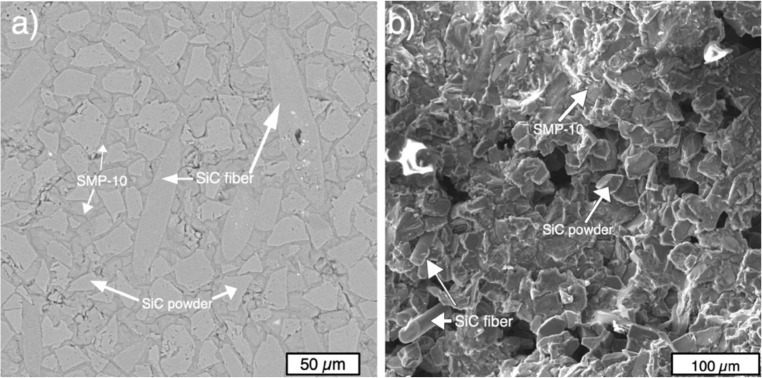
SEM-images of the microstructure (**a**) and fracture surface (**b**) of the SiCf/SiC sample fabricated from irregular particles after six infiltration and pyrolysis cycles.

In the case of the sample fabricated from irregular SiC powders, the microstructure features SiC powder particles with the initial shape and separate SiC fibers. The secondary SiC also formed between powder particles from SMP-10 after pyrolysis (Figure 9a). The pores are also present in the sample, but visually, the sample obtained from irregular particles has a denser structure compared to the sample obtained from spherical particles, which was confirmed by Archimedes measurements. The fracture surface features the initial irregular SiC particles bonded by the secondary SiC, as well as SiC fibers (Figure 9b).

**Figure 10:**
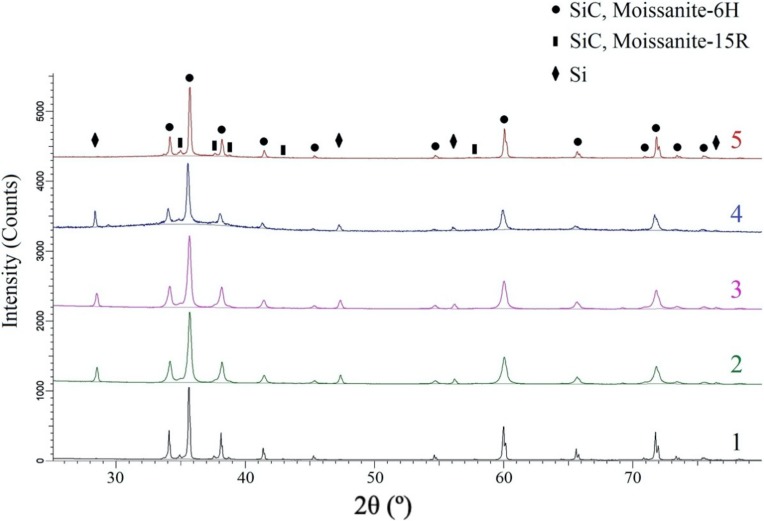
XRD results for the initial SiC powder (**1**), for the powder obtained by spray drying (**2**) and plasma treatment (**3**), and for the samples fabricated by binder jetting followed by SMP-10 infiltration and pyrolysis from spherical (**4**) and irregular (**5**) SiC powders.

Figure 10 shows the XRD results for the initial powders and the fabricated samples. The XRD pattern of the initial powder is characterized by the presence of two SiC modifications: α-SiC peaks with Moissanite-6H and Moissanite-15R crystal structure with an approximate content of 95.6 and 4.4%, respectively. The initial α-SiC powder with Moissanite-6H crystal structure is characterized by the following unit cell parameters: a = 0.3081 nm and c = 1.5114 nm; and Moissanite-15R crystal structure has a = 0.3081 nm and c = 3.7782 nm. The samples 2–4 feature silicon with Fd-3m crystal structure in the amount of 4.4–4.8%. Peak broadening occurred for the powders after spray drying is associated with the coherent scattering region size decrease (down to 50.1 nm).

The ratio of α-SiC content with Moissanite-6H and Moissanite-15R crystal structure in the samples fabricated by binder jetting followed by SMP-10 infiltration and pyrolysis remained the same as in the initial powders. The samples 4 and 5 have the following Moissanite-6H crystal unit cell parameters: a = 0.3080 nm and c = 1.5107 nm, and a = 0.3080 nm and c = 1.5112 nm, respectively, which are close to the reference values for SiC. The coherent scattering region size values are 59.3 nm (for sample 4) and 134 nm (for sample 5). The XRD pattern for sample 4 is characterized by the increased background noise at the peaks’ bottom, suggesting the presence of a small amount of amorphous SiC phase. No phase transformations of silicon carbide were found for different samples.

### 3.4. Mechanical Properties

**Figure 11:**
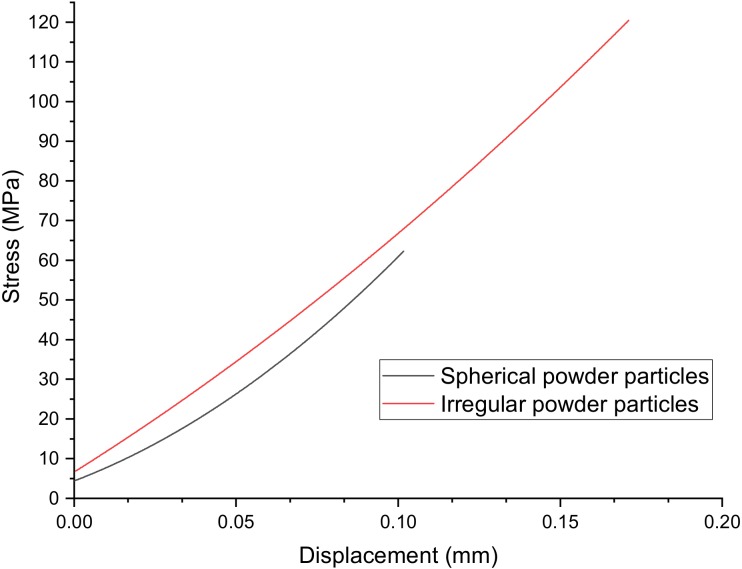
Typical curves for flexural stress-displacement data obtained from the samples produced using spherical and irregular SiC powder particles.

The three-point bending test results showed that the SiC_f_/SiC samples fabricated from the irregular SiC particles have an average flexural strength of 118.7 MPa, while the samples fabricated from the spherical SiC particles have an average flexural strength of 62.7 MPa, as shown in Figure 11. Both materials demonstrate almost linear stress-displacement curves under the flexural load, indicating brittle fracture.

The lower flexural strength values for the samples obtained from spherical particles are the result of a higher pore volume fraction. At the same time, microhardness and fracture toughness are higher for these samples since the separate SiC particles are significantly finer in case of spherical particles, which inhibits crack growth. The measured fracture toughness (shown in Table 2) for the samples from spherical powders is comparable to the values achieved by Hayun et al. [37] for dense (3.18 g/cm^3^) silicon carbide samples fabricated by spark plasma sintering; however, hardness and bending strength values are notably lower. The binder jetted composite SiC_f_/SiC samples showed lower hardness and flexural strength than sintered dense (>98% of the theoretical density) α-SiC material (around 30 GPa hardness and 300 MPa four-point bending strength) [38]. However, the fracture toughness of the binder jetted composite fabricated from the spherical powder is approximately two times higher. The obtained flexural strength values are lower compared to the conventionally fabricated SiC_f/_SiC composites. For example, tape-casted and hot-pressed SiC short-fiber-reinforced SiC composites demonstrated a flexural strength of about 370 MPa [39], but their density was higher, which benefited the mechanical properties. On the other hand, the fracture toughness of the hot-pressed composites was lower (3.23 MPa·m^1/2^) compared to the binder jetted samples. Thus, a further investigation of porosity reduction for binder jetted silicon carbide samples as well as heat treatment effects is necessary to achieve enhanced mechanical properties of the binder jetted SiC_f_/SiC composite. Silicon melt infiltration is one of the possible ways to reduce porosity and increase strength; however, the presence of pure silicon would limit a maximum operating temperature [40]. Another approach to increase the flexural strength of binder jetted SiC_f_/SiC samples might be to increase the volume fraction of SiC fibers and deposit a protective coating on the fibers, which prevents degradation of the fibers and improves the mechanical properties of CMCs as reported in [41,42].

**Figure 12:**
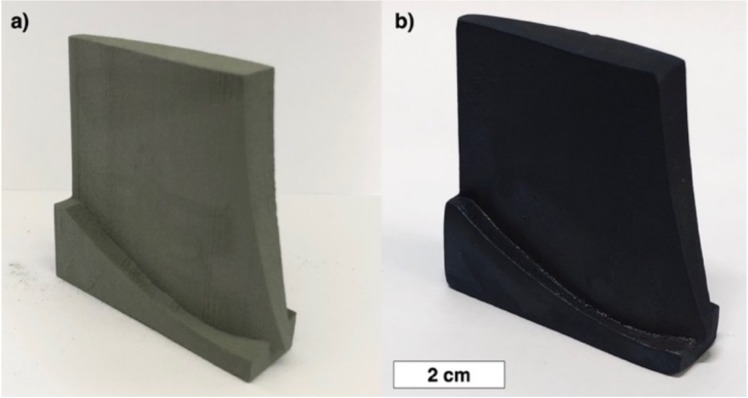
As-fabricated SiC_f_/SiC turbine blade prototype (**a**) and the part after six cycles of infiltration and pyrolysis (**b**).

The presented approach to fabricate SiC_f_/SiC composite parts using binder jetting AM process followed by polycarbosilane infiltration and pyrolysis was demonstrated using a turbine blade prototype model as shown in Figure 12a for as-fabricated green-part and in Figure 12b for the part after six cycles of infiltration and pyrolysis. The binder jetting process followed by several infiltration and pyrolysis cycles has been shown to be a promising method to fabricate SiC_f_/SiC parts enabling complex geometry of parts as well as good dimensional accuracy (about 150–200 µm) and surface finish.

## 4. Conclusions

The present paper demonstrates the feasibility of binder jetting AM of SiC fiber-reinforced SiC composite material followed by polycarbosilane infiltration and pyrolysis. Both irregular and spherical SiC powder particles were used for fabrication of the samples by binder jetting.

The spherical SiC powder particles were obtained by milling the initial irregular SiC powder followed by spray drying and plasma jet treatment. It was demonstrated that the spray drying process followed by thermal plasma treatment can be used for the manufacturing of spherical SiC powder particles with a size of 10–80 µm.

The infiltration and pyrolysis of binder jetted SiC_f_/SiC samples result in a density increase and porosity decrease due to the formation of a secondary SiC phase from SMP-10 polycarbosilane inside the pores. The highest density values (2.52 g/cm^3^ and 2.21 g/cm^3^ for the samples fabricated from irregular and spherical powders, respectively) were achieved after six infiltration and pyrolysis cycles. The difference in the final densities is associated with the presence of submicron pores in spherical powders, which inhibit the further filling of the pores with SMP-10.

The microstructure of the binder jetted samples consists of the initial SiC powder particles that maintained their shape and SiC fibers bonded with a secondary SiC phase.

The three-point bending tests showed that the SiC_f_/SiC samples fabricated from the irregular SiC particles have an average flexural strength of 84.3 MPa, and the samples fabricated from the spherical SiC particles have an average flexural strength of 52.9 MPa. The lower flexural strength values for the samples obtained from spherical particles are the result of higher internal porosity. Microhardness and fracture toughness are higher for the samples fabricated from spherical particles, since the separate SiC particles are significantly finer in this case, which inhibits crack growth.

## Figures and Tables

**Table 1:** Summary of the types of SiC powders and treatments used in the study.

Type of Powder	Powder Treatment	Fibers Used	Fabrication of Samples	Post-Treatment of Samples
Irregular SiC (d_50_ = 38.3 µm)	None (1) Milling; (2) Mixing with 5 wt.% of Si powder; (3) Spray drying; (4) Plasma treatment	30% vol. of SiC fibers mixed with the SiC powder	Binder jetting	SMP-10 infiltration and pyrolysis

**Table 2:** Average values of microhardness and fracture toughness for SiC_f_/SiC samples.

SiC Powder Particles	Hv. GPa	Fracture Toughness K1C, MPa·m^1/2^
Irregular	11.6	3.58
Spherical	20.8	6.13
