# Erratum: Polozov, I., *et al.* Fabrication of Silicon Carbide Fiber-Reinforced Silicon Carbide Matrix Composites Using Binder Jetting Additive Manufacturing from Irregularly-Shaped and Spherical Powders. *Materials* 2020, *13*, 1766

**DOI:** 10.3390/ma13112630

**Published:** 2020-06-09

**Authors:** Igor Polozov, Nikolay Razumov, Dmitriy Masaylo, Alexey Silin, Yuliya Lebedeva, Anatoly Popovich

**Affiliations:** 1 Peter the Great St. Petersburg Polytechnic University, Polytechnicheskaya, 29, St. Petersburg 195251, Russia; n.razumov@onti.spbstu.ru (N.R.); dmasaylo@gmail.com (D.M.); silin8888@mail.ru (A.S.); director@immet.spbstu.ru (A.P.); 2 Federal State Unitary Enterprise “All-Russian Scientific Research Institute of Aviation Materials” State Research Center of the Russian Federation, 17 Radio str., Moscow 105005, Russia; yulia.ananieva@gmail.com

The authors wish to make the following correction to this paper [1]:

Change in Funding.

The authors wish to change the funding part from:

Funding: This research was supported by Russian Science Foundation grant (project No 19-79-30002).

to

Funding: This research received no external funding.

The authors would like to apologize for any inconvenience caused to the readers by these changes.
